# The prevalence of relative age effects in a nationwide analysis of racket sports: Happy birthday?

**DOI:** 10.1371/journal.pone.0316314

**Published:** 2024-12-30

**Authors:** Mert Bilgiç, Alpay Güvenç

**Affiliations:** Department of Coaching Education, Akdeniz University, Antalya, Türkiye; University of Castilla-La Mancha, SPAIN

## Abstract

Relative age effects (RAEs) refer to all consequences of chronological age-based systems. The purpose of this study was to investigate the prevalence of RAEs among Turkish racket sports players. As a nationwide analysis, the present study extends beyond the typical investigations of elite-level popular sports by examining RAEs in racket sports players from the lowest grassroots level to the top and from children to veteran athletes. A total of 57476 racket sports players (i.e., badminton, squash, table tennis and tennis) were evaluated in the study. To investigate interquartile distributions, Chi-square goodness-of-fit tests were used. Odds Ratios (OR) and 95% Confidence Intervals (95% CIs) were calculated to compare quartiles. Poisson regression with canonical link was conducted to analyze the count data. A statistically significant difference in the prevalence of RAEs was noted in both genders and in total sample. The ID in Poisson regression shows that players born at the beginning of the year are 1.63 more likely to be represented than those born at the end of the year. Considering the sports separately, statistically significant distribution bias was found in badminton, table tennis and tennis but not in squash. Moreover, regarding the age categories, the peak RAEs were noted in the youngest age category of tennis as 30.6% of players were in Q1 while only 17.4% were in Q4. Such findings have been discussed with different moderators, hypotheses and models such as the developmental systems model, social agents, psychological issues, and the role of selection processes by coaches. In conclusion, process (i.e. athlete development process) is suggested to be focused instead of a point in the continuum for selection and scouting practices, which may ensure avoiding talent loss and sports drop-out and establishing quality sport participation environments for all.

## Introduction

The competitive nature of sports emphasizes success-oriented practices. Thus, finding diamonds in the rough has been a common concern for sport clubs and any national governing bodies of sport [1, 2]. This perspective has led to the spread of talent programs worldwide to identify and develop “talented” athletes and support them in preparing for the competitive demands of professional sports [2, 3]. Although these programs have been developed to foster athletes’ development, their effectiveness has been criticized because of their scientific foundations such as low predictive value and lack of validity [1]. In addition, preventing talent loss and dropping out are of primary concern in these programs and the whole sporting system [4]. To ensure fair participation, selection, competition and achievement opportunities for each athlete and to prevent talent loss and drop-outs, categorization policy by age is a widely used strategy in sports.

Categorization by chronological age is the most common policy used to group athletes into cohorts based on a certain cutoff date [5, 6]. While a single calendar year is used to form those cohorts in some sports, others may include two or three consecutive calendar years per cohort, particularly in youth sports such as U15, U17 and U20. Although this categorization policy by chronological age is a well-intended approach, in practice there can be inevitably significant (dis)advantages for athletes in the same age cohort from different stands. Considering the categorization policy, January 1st is the most commonly used cutoff date in many sports for cohort organizations [5, 7]. The difference between individuals born in the same year in this chronological age-based system is called “relative age”, and all of the consequences in such systems in terms of performance, success, selection, reselection and/or deselection are called “relative age effects” (RAEs) [8]. The assumed explanation for this effect had nothing to do with astrology but instead was attributed to the fact that athletes are divided into age groups according to their birth date.

Over the last few decades, researchers have investigated the prevalence of the RAEs across several sports from different perspectives [5, 7, 9, 10]. Grondin et al. [11] were the first to report RAEs in ice hockey and volleyball favoring relatively older players and to attribute such results to the categorization system based on a certain cutoff date. However, in almost four decades of research, certain findings show contradictions depending on the nature and demands of sports such as those depending heavily on strength, endurance or technical skills or being an individual or team sport. Moreover, Schorer et al. [12] suggested that competition levels, gender and age categories are strong determinants of RAEs. The greater the physical/endurance demands of a sport are, the greater the number of athletes in that specific sport, the greater the popularity of the sport and the greater the likelihood that the RAEs will exist [7, 10, 12]. RAEs are more prevalent among male athletes than among female athletes [e.g., 13], and specifically the prepubertal period has been mentioned as the critical period in terms of RAEs prevalence [5, 7]. In terms of years, “accumulative advantage” can be a concern here as the attainment of several advantages (e.g., better coaching, training and experience opportunities) in the early years might affect an athlete’s career in the following years [14]. On the contrary, Boccia, et al. [15] stated that being selected for a junior national team is not a reliable marker for future success. Therefore, the continuation of RAEs requires further research considering contradictory perspectives and findings in the literature.

In soccer and ice hockey, which are the most popular sports in RAEs research [9], several findings have confirmed uneven trends favoring early-born athletes [16–18]. Furthermore, Raschner et al. [19] reported the prevalence of RAEs among all athletes involved in strength-, endurance- and technique-related sports in the first winter of the Youth Olympic Games. Similarly, Müller et al. [20] noted the prevalence of RAEs among strength- and endurance-related sports such as ice-hockey and biathlon, but not in technique-related sports such as figure-skating. However, Uğurlu and Bilgiç [21] reported a significantly uneven distribution in men’s singles discipline and males in pairs discipline in figure skating but not in other disciplines, which may indicate that the presence of RAEs differs depending on the demands of each discipline, even in a single sport. Moreover, given that gymnastics is heavily dependent on technique-related skills, Hancock et al. [22] noted a flip flop phenomenon—the reverse of RAEs—favoring relatively younger athletes.

Along with questioning the possible mechanisms of RAEs, the focused sports in the current RAEs literature seem to be another significant concern. As reported by Bilgiç and Işın [9] in a bibliometric analysis, only 281 articles were published on the RAE in journals indexed in WoS (Web of Science) until December 24, 2020, and most of them focused on soccer (n = 97), ice hockey (n = 22), basketball (n = 11) and handball (n = 11). Thus, considering the number of articles in certain sports, racket sports are highly overlooked, with only 7 articles in racket sports (5 in tennis and 2 in table tennis). Soccer, cricket, ice hockey, tennis, volleyball and basketball are stated as the most popular sports in the world considering several paremeters such as participation, having fans and most-watched [23, 24]. Researchers might have a tendency to conduct more research on these sports as well. However, there have been recent studies on RAEs in racket sports, including badminton and padel as less-researched sports, published after this bibliometric analysis by Bilgiç and Işın [9] [e.g., 16–18, 25, 26], they are quite few in number though. For instance, Aku and Yang [26] investigated RAEs in Chinese junior tennis competitions and determined statistically significant over-representation of relatively older players in U12, U14 and U16 age categories, but not in Chinese national female junior tennis team. From a different perspective Delorme et al. [27] investigated RAEs in self-organized sport ractices among adults. Table tennis was the only racket sports investigated in this study with others such as soccer, basketball and swimming, and the results showed no significant RAEs. Furthermore, most studies on RAEs have focused on males while fewer studies have focused on females [7, 26], which also yielded contradictory results [e.g., 10, 28, 29]. Cobley et al. [7] stated in their meta-analysis that only 2% of all participants were females. In a more recent review, Smith et al. [10] reported that RAEs have been a small but consistent influence among female athletes. Additionally, most studies analyzing RAEs are limited to a single sport, which leads us to see an incomplete picture of the whole phenomenon. Only 12.10% (n = 34) of the studies on RAE included multiple sports [9]. As an example of RAEs research including multiple sports, Gil et al [23] investigated RAEs among 9-14-year-old participants in grassroots levels in 37 competitive sports. This study included 500 table tennis, tennis and padel players as a part of 38,381 participants, and RAEs were determined only in 13-year-old male tennis players compared to females. So, there are still many gray areas and gaps to fill to better understand the phenomenon and to reorganize the sporting system accordingly to ensure fair opportunities for all. To the best of our knowledge, there is no such study in the literature that investigated the prevalence of and variations in RAEs among all racket sports players through a nationwide analysis across several factors such as gender, age categories and sports in particular.

Considering the aforementioned points, the purpose of the present study was to investigate the prevalence of RAEs among Turkish racket sports players (i.e., badminton, squash, table tennis and tennis). By conducting a nationwide analysis, the present study extends beyond the characteristics of elite-level popular sports by examining RAEs at the lowest grassroots level from early years to veteran years to ascertain whether there are uneven distributions of RAEs and when it begins and/or ends.

## Materials and methods

### Participants

Badminton, squash, table tennis and tennis were identified as the major racket sports by Lees [30], and this study included participants from these racket sports. A total of 57,476 Turkish racket sports players aged 5 to 85 years (25,464 females, 44.30%) were evaluated in this study. Only players who were active in 2018–2019 season were included in the present study. Of 57,476 players, 17,134 were badminton players (7,849 females and 9,285 males), 13,606 were table tennis players (5,164 females and 8,442 males), 182 were squash players (71 females and 111 males) and 26,554 were tennis players (12,380 females and 14,174 males). The participants were grouped into age categories used by national/international federations of each sport, except for squash. Considering the number of squash players, youth age categories were grouped as one. Further details related to participants were presented in Table 1.

**Table 1:** Descriptives of participants.

Sport	Age Categories	Number of Players	% of Players in the Sport	% of Players in the Whole Sample
**Badminton**	Veterans	345	2.01	0.60
	Adults	1695	9.89	2.95
	U19	1736	10.13	3.02
	U17	1911	11.15	3.32
	U15	3654	21.33	6.36
	U13	3548	20.71	6.17
	U11	4245	24.78	7.39
	Total	17134	100	29.82
**Table Tennis**	Veterans	825	6.06	1.44
	Adults	1205	8.86	2.10
	U19	1322	9.72	2.30
	U15	1484	10.91	2.58
	U13	2545	18.70	4.43
	U11	6225	45.75	10.83
	Total	13606	100	23.67
**Squash**	Veterans	28	15.38	0.05
	Adults	27	14.84	0.05
	Youth	127	69.78	0.22
	Total	182	100	0.32
**Tennis**	Veterans	2466	9.29	4.29
	Adults	796	3.00	1.39
	18U	320	1.21	0.56
	16U	5105	19.22	8.88
	14U	5787	21.79	10.07
	12U	5594	21.07	9.73
	G&O&R	6486	24.43	11.28
	Total	26554	100	46.20

% = Percentage

### Design and procedures

Firstly, the institutional ethical approval was taken from Akdeniz University (Ethical approval number: 2012-KAEK-20/1042).Then, information of racket sports players (i.e., date of birth, gender and sport) was requested from the Ministry of Youth and Sports of Turkey via an offical letter and the dataset was provided in a fully anonymized format (i.e., no names were provided by the ministry) on 18 December 2019 (Approval number: 81091751–130.99-E.1314271). All the procedures conducted in this study were in line with the Declaration of Helsinki.

In accordance with the relevant literature, players were divided into trimesters (Qs) based on their birth months. Since January 1st is the official cutoff date applied in racket sports in Turkey, Q1 included players born from January to March; Q2 included players born from April to June; Q3 included players born from July to September; and Q4 included players born from October to December.

### Statistical analysis

To investigate the RAE, Chi-square goodness-of-fit tests were used to investigate birth month distributions among trimesters. Since there were no available national birth data in Turkey until 2001, the obtained birth month distributions across the trimesters were compared with each other based on the theoretically expected equal distributions, which is in line with previous research [12, 17, 19, 20]. Each age category was evaluated separately and as a whole to determine bias. Furthermore, each analysis was repeated for both genders to investigate possible differences between genders. In order to explain how meaningful the difference, in other words to show practical significance, Cramer’s V was calculated as an effect size calculation. Cramer’s V was interpreted as follows; small (V = 0.06–0.17), medium (V = 0.18–0.29) and large (V ≥ 0.30) [31]. While a large effect size leads to a practical significance interpretation, a small effect size indicates limited practical application. Odds Ratios (OR) and 95% Confidence Intervals (95% CIs) were used to compare quartiles with each other.

Despite the use of Chi-square tests to assess the RAE, which has been widely practiced [18, 32], this approach has been criticized for several reasons (e.g., low statistical power). Thus, Poisson regression with canonical link was conducted to analyze birth count data using the formula y = e(b0+b1x). In this analysis, the birthdate distribution was treated as a continuous variable to investigate the RAE and increase the statistical power by reducing the number of comparisons. Specifically, Poisson regression was conducted to determine how the birth count in a specific week (y) was explained by the time of birth (x). The time of birth (tB) ranging between 0 and 1 was calculated using the formula tB = (WB − 0.5)/52. WB (i.e., week of birth) represents the athletes’ birth week (e.g., in players born between 1st and 7th January WB, it was 1). The relative odds (i.e., index of discrimination, ID) of being selected for a player born in the first week compared to the last week of the competition year. R2 was indirectly calculated from the equation according to previous studies [33, 34]. As in Chi-square tests, analyses were repeated for each sport and gender separately.

All analyses were conducted using IBM SPSS (Version 23.0; IBM, Armonk, NY, USA) and R (Version 4.3.2; R Foundation for Statistical Computing, Vienna, Austria).

## Results

**Fig 1:**
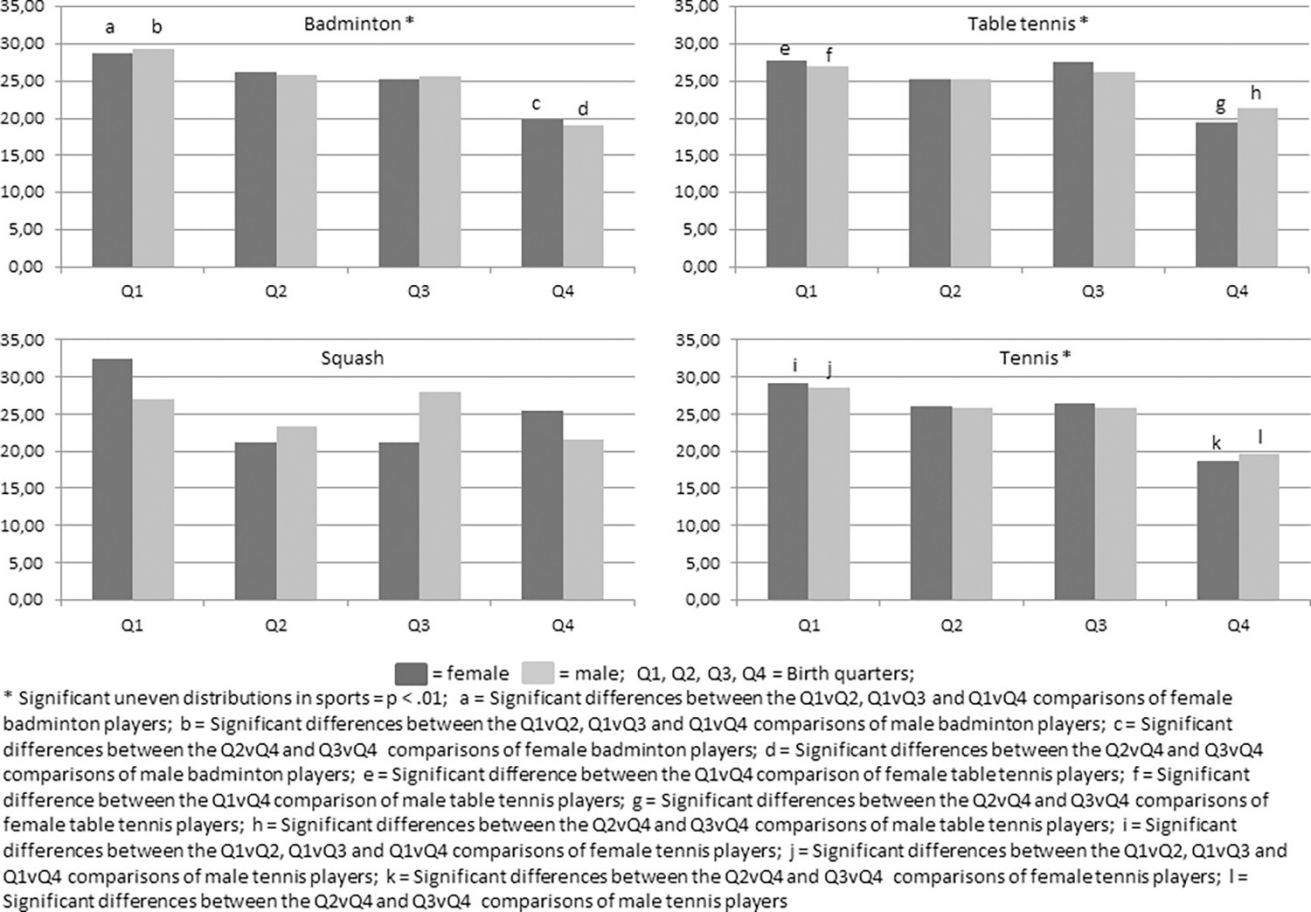
Quarterly percentage distribution of players’ birthdates by sport and gender.

The quarterly distributions of Turkish racket sports players’ birthdates by gender, sport and total are presented in Fig 1 and Table 2. The χ^2^ test results and Cramer’s Vs are also presented, in addition to quarterly comparisons by Odds Ratios (and 95% confidence intervals).

**Table 2:** Quarterly distributions and Odds Ratios (and the 95% confidence interval) of players’ birthdates by gender and sport.

		n	Q1 (%)	Q2 (%)	Q3 (%)	Q4 (%)	χ^2^	*P*	V	Q1vQ2	Q1vQ3	Q1vQ4	Q2vQ3	Q2vQ4	Q3vQ4
**Female**	**All Sample**	25464	7314 (28.7)	6602 (25.9)	6677 (26.2)	4871 (19.1)	516,203	.000*	.08	1.11 (1.06–1.16)^┼^	1.10 (1.04–1.15)^┼^	1.50 (1.43–1.58)^┼^	0.99 (0.94–1.04)	1.36 (1.29–1.43)^┼^	1.37 (1.30–1.44) ^┼^
	**Badminton**	7849	2253 (28.7)	2063 (26.3)	1978 (25.2)	1555 (19.8)	132,902	.000*	.08	1.09 (1.01–1.19)^┼^	1.14 (1.04–1.24)^┼^	1.45 (1.32–1.59)^┼^	1.04 (0.96–1.14)	1.33 (1.21–1.45) ^┼^	1.27 (1.16–1.39) ^┼^
	**Squash**	71	23 (32.4)	15 (21.1)	15 (21.1)	18 (25.4)	2,408	.492	.11	1.53 (0.60–3.91)	1.53 (0.60–3.91)	1.28 (0.52–3.15)	1.00 (0.37–2.67)	0.83 (0.32–2.15)	0.83 (0.32–2.15)
	**Table Tennis**	5164	1437 (27.8)	1308 (25.3)	1419 (27.5)	1000 (19.4)	95,019	.000*	.08	1.10 (0.99–1.22)	1.01 (0.91–1.13)	1.44 (1.29–1.61)^┼^	0.92 (0.83–1.03)	1.31 (1.17–1.46) ^┼^	1.42 (1.27–1.59) ^┼^
	**Tennis**	12380	3601 (29.1)	3216 (26.0)	3265 (26.4)	2298 (18.6)	302,031	.000*	.09	1.12 (1.05–1.20)^┼^	1.10 (1.03–1.18)^┼^	1.57 (1.46–1.68) ^┼^	0.98 (0.92–1.06)	1.40 (1.30–1.51) ^┼^	1.42 (1.32–1.53) ^┼^
**Male**	**All Sample**	32012	9103 (29.3)	8224 (25.8)	8300 (25.7)	6385 (19.1)	495,436	.000*	.07	1.11 (1.06–1.16)^┼^	1.10 (1.05–1.15)^┼^	1.43 (1.36–1.49) ^┼^	0.99 (0.95–1.04)	1.29 (1.23–1.35) ^┼^	1.30 (1.24–1.36) ^┼^
	**Badminton**	9285	2725 (29.3)	2398 (25.8)	2385 (25.7)	1777 (19.1)	202,122	.000*	.09	1.14 (1.05–1.23)^┼^	1.14 (1.06–1.24)^┼^	1.53 (1.41–1.67) ^┼^	1.01 (0.93–1.09)	1.35 (1.24–1.47) ^┼^	1.34 (1.23–1.46) ^┼^
	**Squash**	111	30 (27.0)	26 (23.4)	31 (27.9)	24 (21.6)	1,180	.758	.06	1.15 (0.55–2.44)	0.97 (0.47–2.01)	1.25 (0.59–2.65)	0.84 (0.40–1.77)	1.08 (0.50–2.33)	1.29 (0.61–2.73)
	**Table Tennis**	8442	2290 (27.1)	2129 (25.2)	2218 (26.3)	1805 (21.4)	65,126	.000*	.05	1.08 (0.99–1.17)	1.03 (0.95–1.12)	1.27 (1.16–1.38) ^┼^	0.96 (0.88–1.04)	1.18 (1.08–1.29) ^┼^	1.23 (1.13–1.34) ^┼^
	**Tennis**	14174	4058 (28.6)	3671 (25.9)	3666 (25.9)	2779 (19.6)	248,464	.000*	.08	1.11 (1.04–1.18)^┼^	1.11 (1.04–1.18)^┼^	1.46 (1.37–1.56) ^┼^	1.00 (0.94–1.07)	1.32 (1.23–1.41) ^┼^	1.32 (1.23–1.41) ^┼^
**Total**	**All Sample**	57476	16418 (28.6)	14826 (25.8)	14977 (26.1)	11255 (19.6)	1007,301	.000*	.08	1.11 (1.07–1.14)^┼^	1.09 (1.06–1.13)^┼^	1.46 (1.41–1.51) ^┼^	0.99 (0.96–1.02)	1.32 (1.27–1.36) ^┼^	1.33 (1.29–1.38) ^┼^
	**Badminton**	17134	4978 (29.1)	4461 (26.0)	4363 (25.5)	3332 (19.4)	332,791	.000*	.08	1.12 (1.05–1.18)^┼^	1.14 (1.08–1.21)^┼^	1.49 (1.41–1.59) ^┼^	1.02 (0.96–1.08)	1.34 (1.26–1.42) ^┼^	1.31 (1.23–1.39) ^┼^
	**Squash**	182	53 (29.1)	41 (22.5)	46 (25.3)	42 (23.1)	1,956	.582	.06	1.29 (0.72–2.32)	1.15 (0.65–2.04)	1.26 (0.71–2.25)	0.89 (0.49–1.61)	0.98 (0.54–1.77)	1.10 (0.61–1.97)
	**Table Tennis**	13606	3728 (27.4)	3437 (25.3)	3637 (26.7)	2804 (20.6)	152,970	.000*	.06	1.08 (1.02–1.16)^┼^	1.03 (0.96–1.09)	1.33 (1.24–1.42) ^┼^	0.95 (0.88–1.01)	1.23 (1.14–1.31) ^┼^	1.30 (1.21–1.39) ^┼^
	**Tennis**	26554	7659 (28.8)	6887 (25.9)	6931 (26.1)	5077 (19.1)	546,360	.000*	.08	1.11 (1.06–1.17)^┼^	1.11 (1.05–1.16)^┼^	1.51 (1.44–1.58) ^┼^	0.99 (0.95–1.04)	1.36 (1.29–1.43) ^┼^	1.37 (1.30–1.43) ^┼^

n = number of participants, % = percentage, Q1 = Birth-Quartile 1, Q2 = Birth-Quartile 2, Q3 = Birth-Quartile 3, Q4 = Birth-Quartile 4, χ^2^ = Chi-square, V = Cramer’s V

*p < .01, ┼ Significance of odd ratios

The χ^2^ goodness-of-fit tests revealed statistically significant differences as the overrepresentation of players born shortly after the cutoff date compared to those born shortly before the cutoff date in total and for each sport but squash. The overall distribution bias was slightly greater for females than for males (χ^2^ = 516.203, p < .001, V = .08 and χ^2^ = 495.436, p < .001, V = .07 for females and males, respectively). In particular, the peak RAEs were noted in female tennis players (χ^2^ = 302.031, p < .001, V = .09),. Moreover, significant odds ratios were noted between quartiles. Higher odds ratios were noted between Q1 and Q4, and the peak odds ratio was determined in female tennis players, followed by male badminton players.

**Fig 2:**
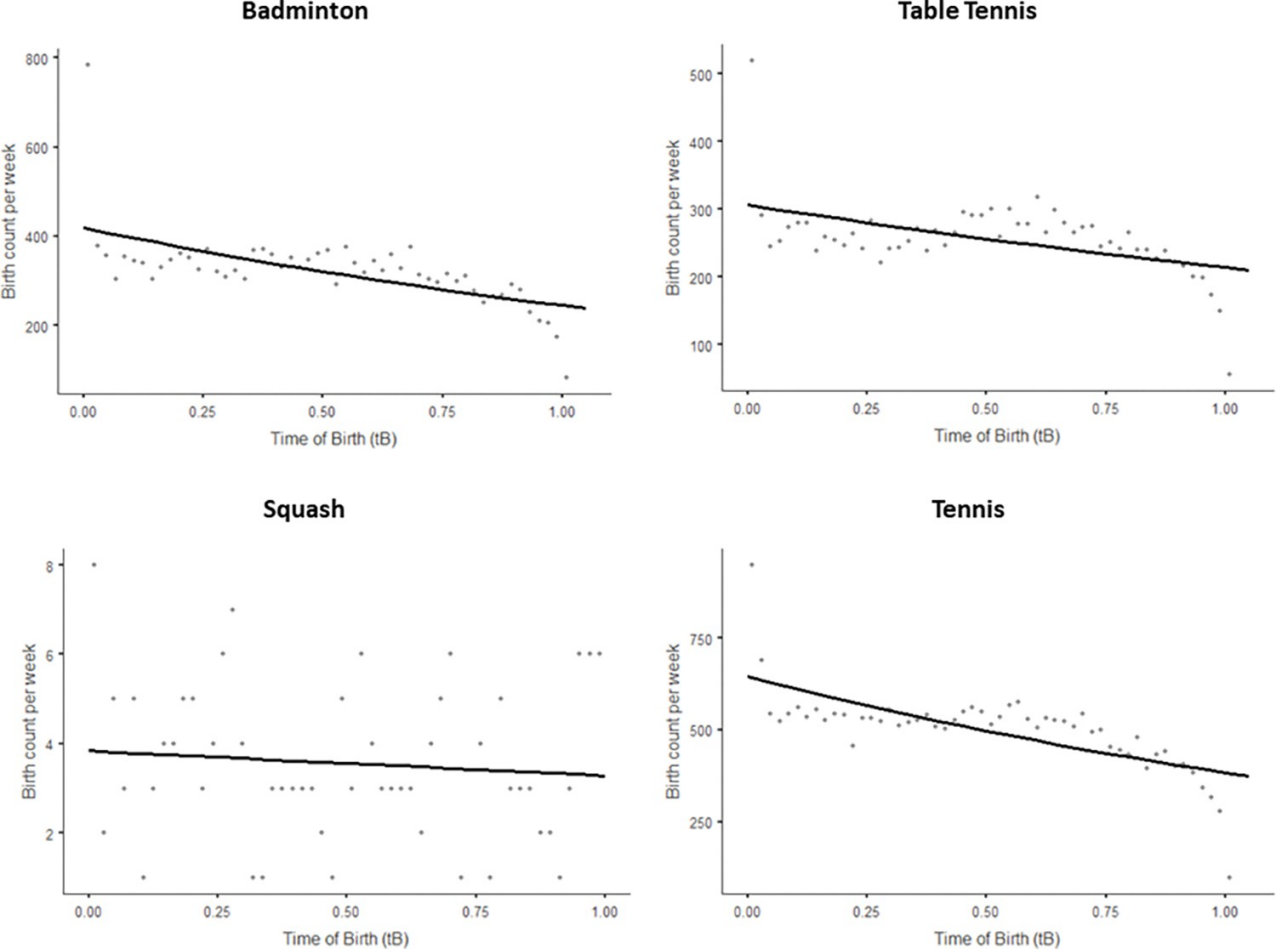
Scatter plots of relative birth frequency by week considering different racket sports. The line represents the best fit of the Poisson regression modelling.

Fig 2 shows scatter plots for the frequency of RAE according to the week of birth for each racket sports. As presented in Table 3, results of the Poisson regression analyses were significant for the total sample (p < .001; R2 = 0.40), and the ID shows that players born at the beginning of the year are 1.63 more likely to be represented than those born at the end of the year. In particular, the Poisson regression showed significant effects for three racket sports (i.e., badminton: p < .001; R2 = 0.39; table tennis: p < .001; R2 = 0.23; tennis: p < .001; R2 = 0.46) but not for squash (p = 0.505; R2 = 0.01). IDs analyses showed that athletes of these three racket sports born at the beginning of the year were more likely to be represented than those born at the end of the year (ID range from 1.43 to 1.71).

**Table 3:** Poisson regression analysis of RAE by gender and sport.

		n	W_B_	T_B_	b_0_	b_1_	ID	R^2^	p
**Female**	**All Sample**	25464	24.70 ± 14.75	0.47 ± 0.28	6.42	-0.51	1.67	0.39	< .001
	**Badminton**	7849	24.70 ± 14.85	0.47 ± 0.29	5.25	-0.51	1.67	0.37	< .001
	**Squash**	71	25.65 ± 16.47	0.48 ± 0.32	0.70	-0.26	1.30	0.02	0.51
	**Table Tennis**	5164	25.13 ± 14.68	0.47 ± 0.28	4.78	-0.42	1.52	0.23	< .001
	**Tennis**	12380	24.52 ± 14.68	0.46 ± 0.28	5.72	-0.56	1.74	0.41	< .001
**Male**	**All Sample**	32012	24.92 ± 14.89	0.47 ± 0.29	6.63	-0.46	1.64	0.39	< .001
	**Badminton**	9285	24.52 ± 14.89	0.46 ± 0.29	5.43	-0.55	1.74	0.37	< .001
	**Squash**	111	25.53 ± 15.43	0.48 ± 0.30	0.87	-0.10	1.11	0.00	0.76
	**Table Tennis**	8442	25.55 ± 14.92	0.48 ± 0.29	5.23	-0.32	1.38	0.20	< .001
	**Tennis**	14174	24.79 ± 14.85	0.47 ± 0.29	5.83	-0.49	1.64	0.46	< .001

n = number of participants, W_B_ = week of birth, T_B =_ time of birth, b_0 =_ intercept term of the exponential model, b_1 =_ coefficient associated with the predictor variable, ID = Index of Discrimination

Table 4 presents the quarterly distribution of racket sports players with χ^2^ test results and Cramer’s Vs by sports and age categories. In addition, comparisons of quartiles are also presented according to Odds Ratios (and 95% confidence intervals).

**Table 4:** Quarterly distributions and Odds Ratios (and the 95% confidence interval) of players’ birthdates by sport and age.

		n	Q1 (%)	Q2 (%)	Q3 (%)	Q4 (%)	χ^2^	*p*	V	Q1vQ2	Q1vQ3	Q1vQ4	Q2vQ3	Q2vQ4	Q3vQ4
**Badminton**	**Veterans**	345	108 (31)	89 (25.6)	83 (23.9)	65 (19.5)	9,448	.024*	.09	1.21 (0.81–1.83)	1.30 (0.86–1.97)	1.66 (1.08–2.55) ^┼^	1.07 (0.70–1.64)	1.37 (0.88–2.12)	1.28 (0.82–1.98)
	**Adults**	1695	482 (28.4)	423 (25)	467 (27.6)	323 (19.1)	36,377	< .001*	.08	1.14 (0.94–1.37)	1.03 (0.86–1.24)	1.49 (1.23–1.81) ^┼^	0.91 (0.75–1.09)	1.31 (1.07–1.60) ^┼^	1.45 (1.19–1.76) ^┼^
	**U19**	1736	512 (29.5)	426 (24.5)	450 (25.9)	348 (20)	31,797	< .001*	.08	1.20 (0.99–1.44)	1.14 (0.95–1.37)	1.47 (1.22–1.78) ^┼^	0.95 (0.78–1.14)	1.22 (1.01–1.49) ^┼^	1.29 (1.07–1.57) ^┼^
	**U17**	1911	590 (30.9)	449 (23.5)	490 (25.6)	382 (20)	47,608	< .001*	.09	1.31 (1.10–1.57) ^┼^	1.20 (1.01–1.43) ^┼^	1.54 (1.29–1.85) ^┼^	0.92 (0.76–1.10)	1.18 (0.98–1.42)	1.28 (1.07–1.54) ^┼^
	**U15**	3654	1077 (29.5)	971 (26.6)	912 (25)	694 (19)	85,628	< .001*	.09	1.11 (0.98–1.26)	1.18 (1.04–1.34) ^┼^	1.55 (1.36–1.77) ^┼^	1.06 (0.94–1.21)	1.40 (1.22–1.60) ^┼^	1.31 (1.15–1.50) ^┼^
	**U13**	3548	993 (28)	935 (26.4)	881 (24.8)	739 (20.8)	40,000	< .001*	.06	1.06 (0.94–1.21)	1.13 (0.99–1.29)	1.33 (1.16–1.52) ^┼^	1.06 (0.93–1.21)	1.25 (1.09–1.43) ^┼^	1.18 (1.03–1.35) ^┼^
	**U11**	4245	1216 (28.6)	1168 (27.5)	1080 (25.4)	781 (18.4)	107,642	< .001*	.09	1.04 (0.93–1.17)	1.13 (1.00–1.27) ^┼^	1.56 (1.38–1.76) ^┼^	1.08 (0.96–1.22)	1.50 (1.32–1.69) ^┼^	1.38 (1.22–1.57) ^┼^
**Table Tennis**	**Veterans**	825	251 (30.4)	203 (24.6)	188 (22.8)	183 (22.2)	13,996	.003*	.08	1.24 (0.95–1.62)	1.34 (1.02–1.75) ^┼^	1.37 (1.05–1.8) ^┼^	1.08 (0.82–1.42)	1.11 (0.84–1.46)	1.03 (0.78–1.36)
	**Adults**	1205	309 (25.6)	289 (24)	336 (27.9)	271 (22.5)	7,744	.052	.05	1.07 (0.85–1.34)	0.92 (0.72–1.15)	1.14 (0.91–1.43)	0.86 (0.69–1.08)	1.07 (0.85–1.34)	1.24 (0.99–1.55)
	**U19**	1322	387 (29.3)	322 (24.4)	323 (24.4)	290 (21.9)	15,011	.002*	.06	1.20 (0.97–1.49)	1.20 (0.97–1.48)	1.33 (1.08–1.66) ^┼^	1.00 (0.80–1.24)	1.11 (0.89–1.38)	1.11 (0.89–1.39)
	**U15**	1484	408 (27.5)	379 (25.5)	392 (26.4)	305 (20.6)	16,792	.001*	.06	1.08 (0.88–1.32)	1.14 (0.93–1.39)	1.34 (1.09–1.64) ^┼^	1.06 (0.86–1.29)	1.24 (1.01–1.53) ^┼^	1.29 (1.04–1.58) ^┼^
	**U13**	2545	687 (27)	643 (25.3)	678 (26.6)	537 (21.1)	22,341	< .001*	.05	1.07 (0.92–1.25)	1.01 (0.87–1.18)	1.28 (1.09–1.5) ^┼^	0.95 (0.81–1.11)	1.20 (1.02–1.40) ^┼^	1.26 (1.08–1.48) ^┼^
	**U11**	6225	1686 (27.1)	1601 (25.7)	1720 (27.6)	1218 (19.6)	102,853	< .001*	.07	1.05 (0.95–1.16)	0.98 (0.89–1.08)	1.38 (1.25–1.53) ^┼^	0.93 (0.84–1.03)	1.31 (1.19–1.46) ^┼^	1.41 (1.28–1.56) ^┼^
**Squash**	**Veterans**	28	6 (21.4)	13 (46.4)	6 (21.4)	3 10.7)	7,714	.052	.30	0.46 (0.11–1.92)	1.00 (0.21–4.67)	2.00 (0.35–11.36)	2.17 (0.52–9.02)	4.33 (0.84–22.23)	2.00 (0.35–11.36)
	**Adults**	27	12 (44.4)	4 (14.8)	7 (25.9)	4 (14.8)	6,333	.096	.28	3.00 (0.61–14.86)	1.71 (0.40–7.43)	3.00 (0.63–14.21)	0.57 (0.11–3.04)	1.00 (0.17–5.75)	1.75 (0.34–8.91)
	**Youth**	127	35 (27.6)	24 (18.9)	33 (26)	35 (27.6)	2,606	.456	.08	1.46 (0.71–2.99)	1.06 (0.53–2.11)	1.00 (0.51–1.97)	0.73 (0.35–1.50)	0.69 (0.34–1.40)	0.94 (0.48–1.87)
**Tennis**	**Veterans**	2466	701 (28.4)	618 (25.1)	629 (25.5)	518 (21)	27,577	< .001*	.06	1.13 (0.97–1.33)	1.11 (0.95–1.30)	1.35 (1.15–1.59) ^┼^	0.98 (0.84–1.15)	1.11 (0.95–1.30)	1.13 (0.97–1.33)
	**Adults**	796	196 (24.6)	197 (24.7)	248 (31.2)	155 (19.5)	21,859	< .001*	.10	0.99 (0.75–1.32)	0.79 (0.60–1.04)	1.26 (0.95–1.69)	0.79 (0.61–1.04)	1.27 (0.95–1.70)	1.60 (1.21–2.12) ^┼^
	**18U**	320	93 (29.1)	66 (20.6)	86 (26.9)	75 (23.4)	5,325	.149	.07	1.41 (0.91–2.19)	1.08 (0.71–1.66)	1.24 (0.80–1.91)	0.77 (0.49–1.20)	0.88 (0.56–1.38)	1.15 (0.74–1.78)
	**16U**	5105	1444 (28.3)	1328 (26)	1320 (25.9)	1013 (19.8)	79,947	< .001*	.07	1.09 (0.98–1.21)	1.09 (0.98–1.22)	1.43 (1.27–1.59) ^┼^	1.01 (0.90–1.12)	1.31 (1.17–1.47) ^┼^	1.30 (1.16–1.46) ^┼^
	**14U**	5787	1620 (28)	1504 (26)	1555 (26.9)	1108 (19.1)	110,429	< .001*	.08	1.08 (0.97–1.19)	1.04 (0.94–1.15)	1.46 (1.32–1.62) ^┼^	0.97 (0.87–1.07)	1.36 (1.22–1.51) ^┼^	1.40 (1.26–1.56) ^┼^
	**12U**	5594	1623 (29)	1441 (25.8)	1452 (26)	1078 (19.3)	112,827	< .001*	.08	1.13 (1.02–1.25) ^┼^	1.12 (1.01–1.24) ^┼^	1.51 (1.35–1.68) ^┼^	0.99 (0.89–1.10)	1.34 (1.2–1.49) ^┼^	1.35 (1.21–1.50) ^┼^
	**G&O&R**	6486	1982 (30.6)	1733 (26.7)	1641 (25.3)	1130 (17.4)	237,031	< .001*	.11	1.14 (1.04–1.26) ^┼^	1.21 (1.10–1.33) ^┼^	1.75 (1.59–1.94) ^┼^	1.06 (0.96–1.16)	1.53 (1.39–1.70) ^┼^	1.45 (1.31–1.61) ^┼^

n = number of participants, % = percentage, Q1 = Birth-Quartile 1, Q2 = Birth-Quartile 2, Q3 = Birth-Quartile 3, Q4 = Birth-Quartile 4, χ^2^ = Chi-square, V = Cramer’s V

*p < .01, ┼ Significance of odd ratios

As presented in Table 4, significant biases were noted among different age categories of racket sports mostly. Adults in table tennis and 18U in tennis were the only two age categories where no significant overrepresentation of any quartile was present (χ^2^ = 7.744, p < .052, V = .05 and χ^2^ = 5.325, p < .149, V = .07, respectively). Moreover, RAEs were more prominent among younger racket sports players considering age categories. The peak RAEs were determined in the Green & Orange & Red Balls age categories in tennis, as 1982 players (30.6%) were in Q1 while 1130 players (17.4%) were in Q4 (χ^2^ = 237.031, p < .001, V = .11). Furthermore, most of the Q1 vs. Q4 comparisons were determined higher compared to other comparisons, and the highest statistically significant odds ratio was determined in Q1 vs. Q4 comparison of players in the Green & Orange & Red Balls age categories in tennis.

## Discussion

The present study investigated the prevalence of and variations in RAEs among Turkish racket sports players across gender, age categories and sports in particular. To the best of our knowledge, this is the first RAEs study including all of the racket sports players in a country by conducting a nationwide analysis with this big data set (i.e., 57,476 racket sports players) and showing the whole picture. The main findings showed a statistically significant distribution bias favoring relatively older players among all racket sports players except for squash players. In terms of gender, RAEs were slightly higher for females than males. Furthermore, younger age categories were more prone to stronger RAEs compared to theolder ones and adults were.

Hypothesizing about the underlying mechanisms regarding the prevalence of RAEs in sports is not straightforward [6]. To gain a thorough understanding and explain RAEs, several models have been proposed. In recent years, Wattie et al. [6] conceptualized a theoretical framework based on Newell’s constraints-based model [35] to explain the phenomenon in depth. Bidirectional interactions among individual (e.g., an athlete’s maturation), task (e.g., type of sport) and environmental (e.g., policies) constraints are suggested along with changes over time as another component affecting each constraint that gives rise to RAEs.

Among the many factors influencing RAEs in youth sports particularly, the growth and maturational advantages of relatively older athletes in the same cohorts are the most prominent and highlighted concerns [36]. Maturational-selection hypothesis is prominently supported and points to the early attainments of relatively older athletes through benefitting their physical advantages compared to their relatively younger counterparts [7, 10, 37]. In the present study, the highest determined RAEs were mostly in the years referring to prepubertal and pubertal years, which are characterized by periods of strength development, changes in aerobic power and peak height velocity, as suggested by Beunen and Malina [38]. The findings of the present study fit in the literature considering the time and tempo differences at which such changes occur [39], and the advantages of better endurance, muscular strength, aerobic power and speed in many sports [40]. Thus, the prevalence of relatively older youth racket sports players in the present study seems to be expected, and these aforementioned advantages are hypothesized to be strengthened further through Pygmalion effects, Galatea effects and Matthew effects [41], which extends the discussions on the RAE to social agents, psychological issues, and the role of selection processes by coaches. This formation, which is highly affected by RAEs, may give rise to greater differences between individuals with regard to certain attainments such as selection, reselection, success, quality equipment, quality training/coaching and experience [42]. Thus, the future careers of youth athletes in the coming years as adults might be formed through those attainments and/or deficiencies. Questioning the continuation of RAEs by age seems quite important for coaches and governing bodies of sports to better manage talent identification and development processes and to avoid drop-outs. From this perspective, RAEs seem to have considerable impacts on athletes’ career pathways, drop-out rates and quality of sport [43]. In the present study, RAEs were still evident at the adult and veteran levels in all racket sports, except for squash, following the youth categories. Such results might be affected by the advantageous position of early-born players in terms of the aforementioned attainments, which may denote an “accumulative advantage” [14].

However, the findings in squash, as an exception, should be reconsidered in this context. No RAEs were noted in any age group (i.e., youth, adults and veterans) in squash, yet the popularity and public attention of squash in Turkey seems to be a strong moderator of such findings. Compared to other sports, squash is the least popular racket sport, as is also understood from the number of players. While the other three racket sports (i.e., badminton, table tennis and tennis) have their own sports federations, squash is governed by Developing Sports Federation of Turkey which includes nine other sports as well such as cricket and korfball. Considering the number of coaches in these four sports, there were only 3 certified squash coaches in Turkey in 2018 while the numbers were 726, 352 and 1273 for badminton, table tennis and tennis coaches, respectively (https://shgm.gsb.gov.tr/Sayfalar/175/105/Istatistikler). This denotes that contextual factors might play significant roles in terms of the prevalence of RAEs, which points to the proposition by several studies that the prevalence of RAEs is higher in popular sports in certain settings [e.g., 5, 7, 10]. Gil et al. [23] discussed this situation as in sports with less attendance and popularity, each individual willing to participate might have the opportunity to do so, regardless of their relative age, while in sports with higher popularity, relatively younger participants may face some challenges to navigate several obstacles. Considering the presence and prevalence of RAEs, the characteristics and demands of a sport can be considered the other highly important subjects to concentrate on while understanding the underlying mechanisms involved. In the relevant literature, no or reverse RAEs have been discussed in sports that are heavily dependent on technical skills [e.g., 12, 44]. In particular, table tennis is suggested to be a skill/technique-dominant sport compared to other sports such as track and field, alpine skiing and tennis [44, 45]. Romann and Fuchslocher [44] noted that RAEs favor early-born tennis players while reverse RAEs were found in table tennis players, and they discussed these findings by pointing out the different demands of each sport such as strength and technique. However, Faber et al. [45] showed uneven representation favoring early-born table tennis players in the U15 world and Europe rankings and U15 and U18 rankings in France, but no such effect was reported in older age categories or among veterans. On the other hand, in the present study, RAEs favoring early-born players were determined in each racket sport and age category, except for 18U tennis, adult table tennis and squash. The findings in badminton and tennis are in line with the relevant literature to a certain extent, and considering the characteristics of both sports in terms of strength- and endurance-related demands, the aforementioned hypotheses can be suggested as explanations for such findings. For instance, in tennis, Gerdin et al. [46] noted that RAEs in Swedish tennis have similar patterns indicating the advantageous positions of early-born players. Zháněl et al. [18] also noted significant RAEs in the top 100 elite female tennis players in 2007–2016 but not in the 17–18-year-old group, which is quite similar to our finding that RAEs were not evident in 18U tennis players. Similar studies have been conducted that highlight the advantages of relatively older tennis players, both in juniors and adults [16, 26, 44]. Such findings might support the proposition by Kovacs [47] that tennis has evolved from a sport dominated by technical/tactical skills to a sport highly dominated by physical skills and fast serves. In badminton, Bilgiç and Devrilmez [17, 32] noted RAEs in European badminton both in terms of participation trends and medal owners. However, earlier studies revealed no such significant differences in badminton [e.g., 48, 49], and Romaneiro et al. [50] reported a significant difference in RAEs only among female badminton players who participated in the 2008 Beijing Olympic Games. Such findings also bring similar questions to mind in terms of the changing characteristics of the game, as in the proposition by Kovacs [47] in tennis. However, the findings in table tennis both in the literature and in the present study are controversial to a certain extent, which leads to the need for further research on table tennis to obtain a thorough understanding.

Similar to table tennis, the female context in RAEs studies is another subject that needs further research considering the contradictory findings and the number of studies. RAEs have shown stronger trends in male athletes, and the number of studies in males is higher than that in females [e.g., 7, 10, 26, 29, 48]. Cobley et al.’s [7] meta-analysis revealed that only 2% of participants who were investigated for RAEs were females and that there was a greater uneven distribution in males than in females. Raschner et al. [19] observed similar results among participants in the Youth Olympic Games 2012. Recently, Reed et al. [51] reported lower RAEs in girls than in boys in school sports, and Smith et al. [10] noted a significant but small RAEs in females. The findings of the present study revealed RAEs in both males and females. In particular, stronger RAEs were noted for female tennis and table tennis players while the opposite was the case for badminton players (i.e., a stronger RAE for male badminton players). Similar findings were presented in the relevant literature to a certain extent. Considering racket sports, Bilgiç and Devrilmez [17, 32] reported RAEs for both males and females among European badminton players; however, stronger RAEs were determined in males considering participation in European Championships [17] while stronger RAEs were noted in females than males considering reaching the podium [32]. Thus, it should be highlighted that there are several moderators and constraints affecting the prevalence of RAEs. In tennis, Gerdin et al. [46] pointed out similar findings that the existence of RAEs was more prevalent in females than in males, yet it appeared to be significantly more prevalent in both genders. Romann and Fuchlocher [44] reported significant RAEs in female tennis players favoring early-borns, while reverse RAEs were observed in female table tennis players. Thus, along with gender, several other concerns require further research, and there seem to be strong constraints to reconsider.

### Practical applications and future directions

This study shows the biased distribution of racket sports players in Turkey which conveys a strong message to coaches, scouts and policy makers. These results can help them to gain a comprehensive understanding of RAEs in both elite and grassroots levels, and urge them to take action to ensure fair and equal opportunities for participation, competition and achievement for all players. They should think twice while deciding on athletes’ “talents” during their selection and reselection/deselection practices. Future initiatives are suggested to increase awareness through organizing meetings, seminars, workshops and/or briefing of coaches, scouts and policy makers on RAEs in depth and how it affects the sporting system, particularly talent identification and development process of youth athletes. Coaches are known as important decision-makers while creating the teams along with scouts who are talent evaluators, travelling and/or following certain players and evaluating their set of skills and talents. So, making them aware of the current trends and the results is quite crucial. Thus, organizing workshops and seminars to deliver such concepts as RAEs, long term athlete development, growth and maturation to them in order to increase their awareness can be very useful. They should be encouraged to run talent-related issues as a process instead of a point in the continuum, to take all these factors into consideration while deciding on players, particularly during prepubertal and pubertal years. Thus, they should act cautiously. For policy makers, modifications of the play system, which is based on chronological age currently, can be a concern to take an action. In particular, increasing policy makers’ awareness on related subjects might engage them to consider alternative systems such as bio-banding discussed by Malina et al. [52]. Bio-banding is a biological-age focused system and was applied in several settings such as soccer [53], cricket [54] and handball [55]. All of them suggested it as an alternative to chronological-age system in order to enhance talent development of youth athletes and to address specific developmental needs of players at different stages of their development. Furthermore, bithday banding was another strategy, suggested and applied in England Squash by Kelly et al. [56] which showed no significant difference between quartiles in contrast to several RAEs studies, including ours. This strategy seems easier to apply in the field compared to bio-banding, thus can be suggested as a useful tool for policy-makers to moderate RAEs. This study calls their attention for taking action to avoid talent loss and sports drop-out, and to establish quality sport participation environments for all.

## Conclusions

This study aimed to investigate RAEs in Turkish racket sports settings. The obvious strength of the present study is that it analyzed complete nationwide data comprising all of the active racket sports players throughout the season in Turkey. However, not every sport receives the same public attention in Turkey, which should be considered while interpreting the findings and their generalizability. Significant RAEs were determined in both genders and in the total sample. Considering the racket sports, significant prevalence of RAEs were noted in badminton, table tennis and tennis, but not in squash, with differences among age categories. Several moderators of this effect such as developmental age, age-grouping policies, and the popularity of sports have been discussed within the proposed developmental systems model proposed by Wattie et al. [6]. This study has some limitations as well. Firstly, there might be several other factors contributing RAEs such as socio-economic backgrounds and access to training facilities. Although this study was conducted with a large group of players, which makes this study very strong in terms of its generalizability to Turkish racket sports context, other possible factors could not be discussed due to the limited data availability. Considering the cross-sectional design of the study on all racket sports players in Turkey, future research is suggested with longitudinal designs to show any changes in specific sports in time, to capture players’ career trajectories in years and to show whether an accumulated advantage is the case for racket sports or not in terms of selection and reselection/deselection patterns. Accepting equal distribution theoretically is another limitation of this study, which is due to the lack of birthdata in the country. Overall, future research is needed to obtain a thorough understanding of the phenomena considering the missing parts, and there is a need to focus on finding solutions to mitigate RAEs.
